# A +1 ribosomal frameshifting motif prevalent among plant amalgaviruses

**DOI:** 10.1016/j.virol.2016.07.002

**Published:** 2016-11

**Authors:** Max L. Nibert, Jesse D. Pyle, Andrew E. Firth

**Affiliations:** a Department of Microbiology & Immunobiology, Harvard Medical School, Boston, MA 02115, USA; b Harvard Ph.D. Program in Virology, Division of Medical Sciences, Harvard University, Boston, MA 02115, USA; c Division of Virology, Department of Pathology, Addenbrooke’s Hospital, University of Cambridge, Cambridge CB2 0QQ, UK

**Keywords:** Amalgaviridae, Coiled coil, Database mining, dsRNA virus, Fungal virus, Plant virus, Ribosomal frameshifting

## Abstract

Sequence accessions attributable to novel plant amalgaviruses have been found in the Transcriptome Shotgun Assembly database. Sixteen accessions, derived from 12 different plant species, appear to encompass the complete protein-coding regions of the proposed amalgaviruses, which would substantially expand the size of genus *Amalgavirus* from 4 current species. Other findings include evidence for UUU_CGN as a +1 ribosomal frameshifting motif prevalent among plant amalgaviruses; for a variant version of this motif found thus far in only two amalgaviruses from solanaceous plants; for a region of α-helical coiled coil propensity conserved in a central region of the ORF1 translation product of plant amalgaviruses; and for conserved sequences in a C-terminal region of the ORF2 translation product (RNA-dependent RNA polymerase) of plant amalgaviruses, seemingly beyond the region of conserved polymerase motifs. These results additionally illustrate the value of mining the TSA database and others for novel viral sequences for comparative analyses.

## Highlights

• A number of new plant amalgavirus sequences have been found in the TSA database.
• They provide support for a prevalent +1 frameshifting motif in amalgaviruses.
• A variant motif is identified in a subset of these viruses from related plants.
• The ORF1 product of amalgaviruses has propensity to form α-helical coiled coil.
• The TSA database is a useful source of new viral sequences for comparative analyses.

## 1 Introduction

Family *Amalgaviridae* is a recently recognized taxon that currently comprises four species of plant viruses (*Blueberry latent virus*, *Rhododendron virus A*, *Southern tomato virus*, *and Vicia cryptic virus M*) in one genus (*Amalgavirus*) (Adams et al., 2014, Liu and Chen, 2009, Martin et al., 2011, Sabanadzovic et al., 2009, Sabanadzovic et al., 2010). These plant amalgaviruses have small dsRNA genomes (3427–3437 bp) and have not yet been shown to form *bona fide* virions. Instead, they are transmitted vertically through seeds and are thought unlikely to be capable of efficient extracellular transmission, unless possibly by vector. The genomic plus strands of plant amalgaviruses encompass two partially overlapping long open reading frames (ORFs), with downstream ORF2 overlapping ORF1 in the +1 frame. They are thereby thought to encode only two proteins, an ORF1-encoded product of unknown specific function (though potential icosahedral capsid protein (CP), filamentous nucleocapsid (NC) protein (Krupovic et al., 2015), or replication factory matrix-like protein (Isogai et al., 2011)) and an ORF1+2-encoded fusion protein that is translated consequent to +1 programmed ribosomal frameshifting (PRF) (Depierreux et al., 2016, Firth et al., 2012, Liu and Chen, 2009, Martin et al., 2011, Sabanadzovic et al., 2009, Sabanadzovic et al., 2010). The ORF2-encoded portion of this fusion protein is indicated by conserved sequence motifs to be the viral RNA-dependent RNA polymerase (RdRp).

For the current report, we undertook studies to identify novel plant amalgavirus sequences, with the goal of learning more about these viruses through sequence comparisons. Liu et al. (2012) searched the Expressed Sequence Tags (EST) database at GenBank/EMBL/DDBJ for amalgavirus-like sequences and identified partial sequences (268–2127 nt in length) from 7 different plant species. We searched instead the Transcriptome Shotgun Assembly (TSA) database at GenBank/EMBL/DDBJ in an effort to identify more complete sequences. Here we report the complete protein-coding sequences of 16 proposed new amalgaviruses, derived from 12 different plant species, plus the nearly complete protein-coding sequences of 3 others. Detailed examinations of these sequences provided several new insights as described below.

## 2 Results

Using the predicted ORF1+2-encoded fusion protein sequence of blueberry latent virus (BLV) (GenBank YP_003934623) as query for a tblastn search of the TSA database for plants (NCBI taxonomic identifier 3193), we identified 37 TSA accessions with E-value scores of 0.0, indicating strong sequence similarities, and lengths between 2793 and 3478 nt, approximating the genome lengths of previously characterized plant amalgaviruses (Table 1, bottom). Some of the E=0.0 accessions derived from the same plant species (*Allium cepa* and *Lolium perenne*) and were nearly identical to one another (≥99% identity), so that after the shorter among these replicates were also excluded, we were left with a set of 19 distinct TSA accessions for further study (Table 1, top). Using the predicted ORF1+2-encoded fusion protein sequences of the other previously characterized plant amalgaviruses as queries in tblastn searches of the TSA database for plants did not expand this list of E=0.0 accessions.

**Fig. 1:**
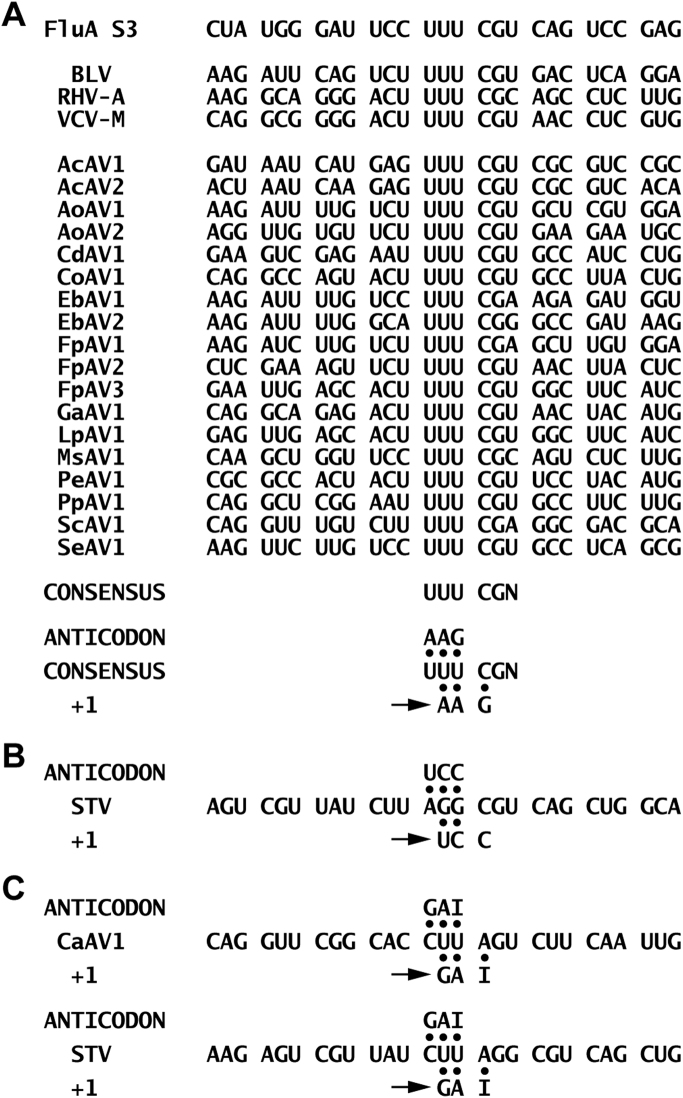
Motifs for +1 PRF. Anticodon:codon base pairs are indicated by filled circles. The positions of these +1 PRF motifs in a broader, aligned RNA sequence context are shown in Fig. S3. (A) Previously identified motif from influenza (Flu)A virus segment (S)3 and previously proposed motifs from plant amalgaviruses BLV, RHV-A, and VCV-M (Firth et al., 2012) are shown. Proposed motifs from newly proposed plant amalgaviruses are also shown, along with the consensus at bottom. Both UUU and UUC are decoded by a single tRNA^Phe^ iso-acceptor that has anticodon 3′AAG (Grosjean et al., 2010). First positioned on codon UUU in the +1 PRF motif, this tRNA is then thought to slip forward by one nucleotide (arrow) in the P site (onto codon UUC), positioning the next codon (GNN) in the A site for continued translation. (B) Previously proposed motif from plant amalgavirus STV (Depierreux et al., 2016) is shown. Anticodon 3′UCC (first positioned on codon AGG in the motif), was suggested to slip forward by one nucleotide in the P site (onto codon GGC), positioning the next codon (GUC) in the A site for continued translation. (C) Newly proposed motifs from plant amalgaviruses CaAV1 and STV are shown. Anticodon 3´GAI (first positioned on codon CUU in the motif) is thought to slip forward by one nucleotide in the P site (onto codon UUA), positioning the next codon (GNC) in the A site for continued translation.

Do these 19 TSA accessions represent the nearly complete genome sequences of novel plant amalgaviruses? Strikingly, as in previously characterized plant amalgaviruses, the apparent plus-strand sequence of each of these accessions contains two partially overlapping long ORFs, with downstream ORF2 overlapping ORF1 in the +1 frame. The lengths of the ORF1–ORF2 overlap regions in the sequences range from 287 to 968 nt, compared with 293–611 nt in previously characterized plant amalgaviruses. Also strikingly, in the overlap regions of the sequences except the one from *Capsicum annuum,* and positioned in the proper reading frame in each sequence, is found the putative +1 PRF motif UUU_CGN (underline, codon boundary for ORF1; N, any nucleotide; CGN, a rare Arg codon) (Fig. 1A), which has been shown to promote translation of the influenza A virus PA-X protein (Firth et al., 2012, Jagger et al., 2012) and also recently proposed to allow ORF1+2-encoded fusion protein translation by plant amalgaviruses (Firth et al., 2012) and the amalga-like mycovirus Zygosaccharomyces bailii virus Z (ZbV-Z) (Depierreux et al., 2016). This finding suggests to us the strong likelihood that the ORF2 product encoded by each of the 19 TSA accessions is translated as part of an ORF1+2-encoded fusion protein consequent to +1 PRF at the position of the proposed motif (Fig. 1A). The proposed motif for +1 PRF in the TSA accession from *C. annuum* is analyzed in Discussion.

As we were performing the preceding analysis, we noted that in 7 of the 19 TSA accessions, ORF1 and/or ORF2 remains open to the respective nucleotide sequence terminus (i.e., is not flanked by one or more stop codon) and encodes a smaller-than-expected protein product (Table 1, top). These 7 sequences hence appear to be partially truncated with respect to their protein-coding regions. In an effort to correct this situation, we turned to data sets in the Sequence Read Archive (SRA) database at NCBI, which were accessible for each of these TSA accessions. By examining the SRA data sets and incorporating additional reads into the transcript contigs, we were able to extend the lengths of 5 of the TSA accessions (GenBank GAYX01076418, GBXZ01009138, GCJW01039808, GEAC01063629, and GECO01025317), for 4 of them such that their protein-coding regions are no longer truncated (Table 1, top). As a result, the protein-coding regions of only 3 of the 19 TSA accessions appear to remain truncated at one or both termini (GenBank GAMH01005363, GBIE01028534, and GECO01025317). See Table S1 for reassembly information for the 5 extended sequences and Data S1 for the reassembled sequences themselves.

Table 1 includes the protein lengths of the ORF1-, ORF2-, and ORF1+2-encoded translation products deduced from the 19 TSA-derived amalgavirus-like sequences as well as from the four originally characterized plant amalgaviruses. Notably, the ORF1-, ORF2-, and ORF1+2-encoded protein lengths deduced from the 16 sequences that encompass complete protein-coding regions span narrow ranges (ORF1p, 375–403 aa; ORF2p post-frameshifting sequences, 769–787 aa; ORF1+2p, 1048–1071 aa), very similar to those spanned in the original plant amalgaviruses (ORF1p, 375–404 aa; ORF2p post-frameshifting sequences, 771–789 aa; ORF1+2p, 1054–1077 aa) (Table 1). These protein lengths deduced from the other 3 TSA-derived amalgavirus-like sequences are generally smaller, consistent with their partial truncation at one or both ends, probably due to incomplete sequencing.

**Fig. 2:**
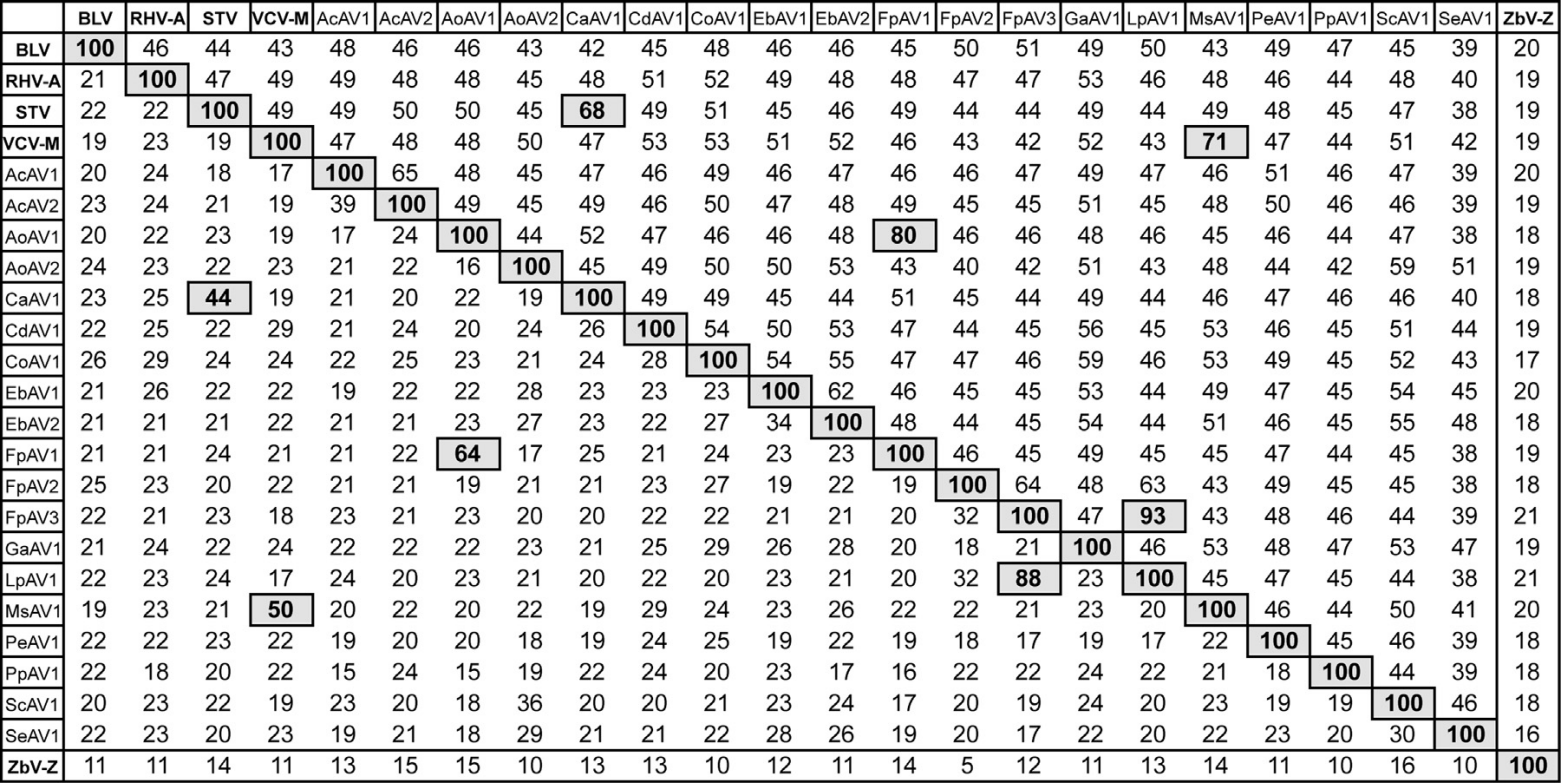
Pairwise sequence identity scores. Sequences of the ORF1 (lower left) and ORF2 (upper right) translation products of the indicated viruses (original and proposed) were compared in pairs using EMBOSS: needle or needleall. Sequence identity scores are shown in %. Shading off the diagonal highlights more closely related pairs for which the ORF1p score is >40% and the ORF2p score is >65%. For these analyses, the ORF1p sequences of AoAV1 and PpAV1 began with the first residue instead of the first Met residue since their encoding sequences appear to be 5′-truncated, and the ORF2p sequences of AoAV1 and SeAV1 ended with the last residue instead of the last residue before the downstream stop codon since their encoding sequences appear to be 3′-truncated; as a result, their scores here may be artificially low in some instances.

When the 19 deduced ORF2p sequences were used as queries in PSI-BLAST searches of the Non-redundant Protein Sequences (NR) database, each was found to be highly similar to the ORF2p (RdRp) sequences of originally characterized plant amalgaviruses (E-values, 0.0). As another way to address the degrees of similarity among these proposed and original plant amalgaviruses, we performed pairwise alignments. The pairwise identity scores for their separate ORF1 and ORF2 products are shown in Fig. 2 and provide further evidence that they are all closely related, especially as reflected by the scores for ORF2p (RdRp). Some pairs are especially closely related, namely, Capsicum annuum amalgavirus 1 (CaAV1) and STV, MsAV1 and VCV-M, AoAV1 and FpAV1, and FpAV3 and LpAV1 (See Table 1 for other abbreviations). Interestingly, in each of these four pairs, the sequences originated from plants of the same taxonomic family and subfamily: CaAV1 and STV, *Solanaceae*/*Solanoideae*; MsAV1 and VCV-M, *Fabaceae/Faboideae*; AoAV1 and FpAV1, *Poaceae/Pooideae*; and FpAV3 and LpAV1, *Poaceae/Pooideae*. These latter findings are consistent with coevolution of amalgaviruses with their respective plant hosts.

**Fig. 3:**
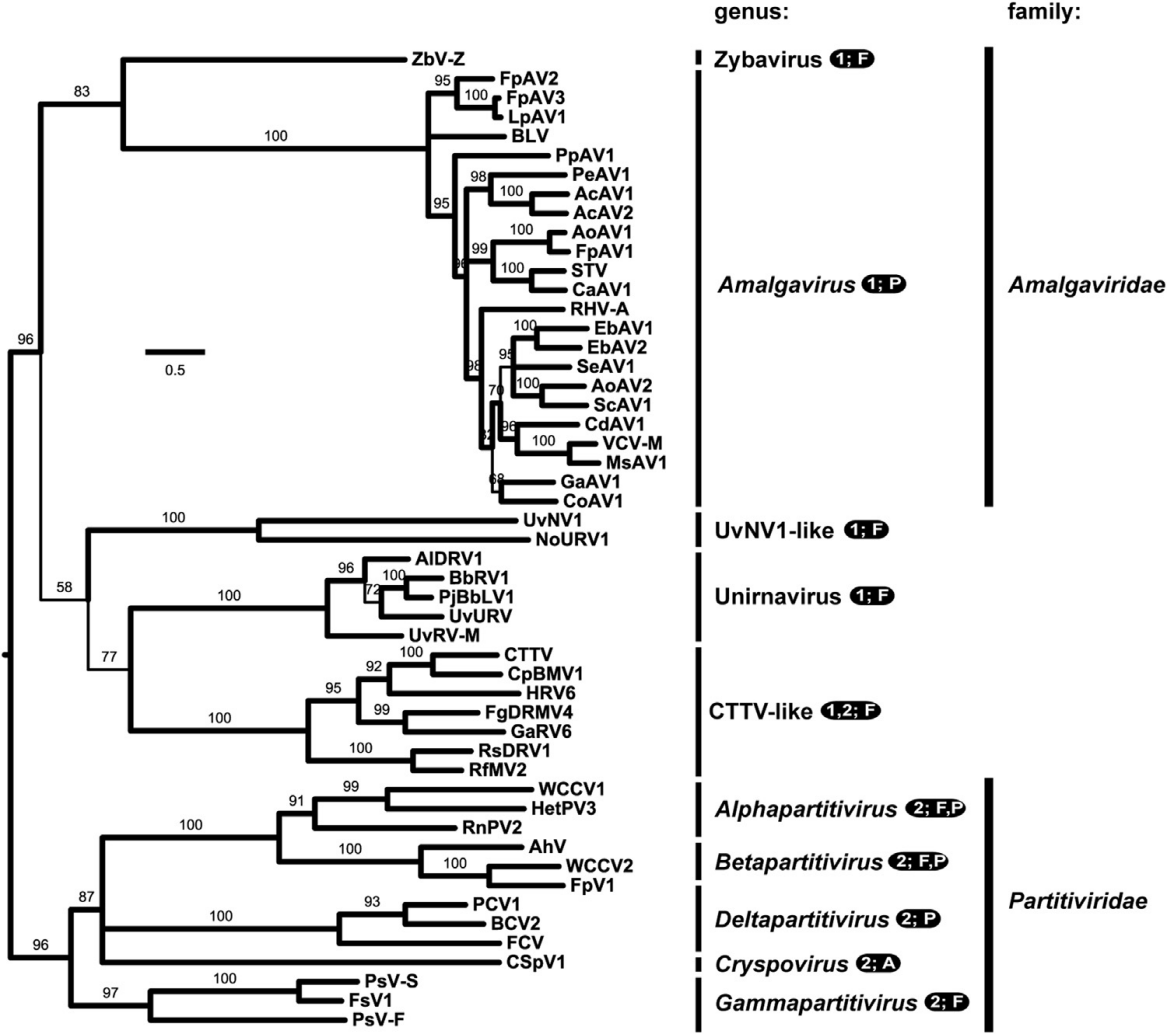
Phylogenetic tree, ORF2p (RdRp). Sequences of the ORF2 translation products were aligned using MAFFT and then subjected to phylogenetic analysis using PhyML as described in Materials and Methods. Values estimated from the data were Proportion of invariable sites, 0.010, and Gamma shape parameter, 1.473. Alternative use of the rtREV amino acid substitution model for PhyML (in place of LG) yielded results largely identical to those shown here. Proposed plant amalgaviruses new to this report are labeled in gray. The tree is displayed as a rectangular phylogram rooted on the branch to family *Partitiviridae* members. Branch support values are shown in %, and those with support values <50% are collapsed to the preceding node. The few branches with support values between 50% and 80% are drawn with thinner lines. Scale bar, average number of substitutions per alignment position. See Table S2 for a summary of abbreviations and GenBank numbers. Vertical lines: approved or proposed spans of genera and families (family *Amalgaviridae* has been proposed to encompass proposed genus Zybavirus by Depierreux et al. (2016)). For each genus-level taxon, the number of characterized genome segments for each virus (1 or 2) and known hosts (P, plants; F, fungi; A, alveolate protist) are indicated.

The 19 deduced ORF2p (RdRp) sequences were next compared by phylogenetic methods. The sequence set for these studies included not only the proposed and original plant amalgaviruses but also a number of viruses whose RdRp sequences have been previously noted to be related to them: ZbV-Z (Depierreux et al., 2016), monosegmented viruses from proposed genus Unirnavirus (Jiang et al., 2015, Koloniuk et al., 2015, Kotta-Loizou et al., 2015, Lin et al., 2015, Nerva et al., 2015, Zhu et al., 2015); viruses related to CTTV, which are presumably all bisegmented (Botella et al., 2015, Márquez et al., 2007, Vainio et al., 2012, Yu et al., 2009, Zheng et al., 2013); and representative bisegmented viruses from family *Partitiviridae* (Nibert et al., 2014) (see Table S2 for abbreviations and GenBank numbers for the additional viruses; RdRp is generally encoded on RNA1 of the bisegmented viruses). Sequences were aligned using MAFFT (Katoh et al., 2013) and then used for maximum-likelihood phylogenetic analyses using PhyML (Guindon et al., 2010) with the LG or rtREV substitution model for amino acids. The resulting RdRp-based trees provided consistent strong evidence that the proposed and original plant amalgaviruses all cluster together in the same taxon (Fig. 3), corresponding to approved genus *Amalgavirus*. Amalga-like mycovirus ZbV-Z is next most closely related to this taxon (Fig. 3), consistent with previous findings (Depierreux et al., 2016, Koloniuk et al., 2015).

**Fig. 4:**
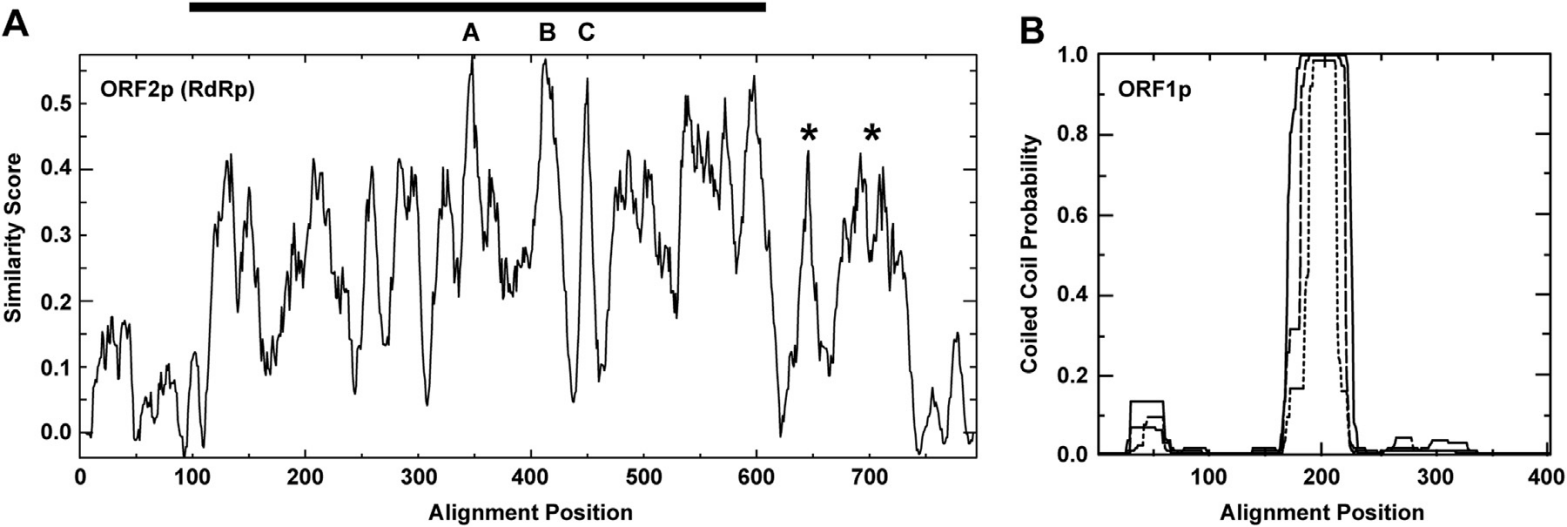
Graphical analyses, ORF2p (RdRp) and ORF1p. (A) The ORF2p (RdRp) alignment for plant amalgaviruses shown in Fig. S1 was analyzed using EMBOSS: plotcon, with a window size of 10 for averaging the similarity scores. Labels A, B, and C indicate peaks corresponding to those respective RdRp motifs. The horizontal line at top indicates the span of homologies to picornavirus RdRps identified by hhpred, as implemented with defaults at http://toolkit.tuebingen.mpg.de/hhpred. Asterisks identify peaks corresponding to highly conserved sequences in a C-terminal region seemingly outside the conserved core RdRp region. (B) The ORF1p alignment for plant amalgaviruses shown in Fig. S2 was analyzed using PCOILS. Results are shown for averaging windows of 14 (dotted line), 21 (dashed line), and 28 (solid line). Fig. S2 also highlights the regions of coiled coil propensity predicted for each individual virus. Graphical results for a representative individual plant amalgavirus sequence (STV) and others are shown in Fig. S4.

Multiple sequence alignments for ORF2p from proposed and original plant amalgaviruses were also examined in detail for conserved residues including known RdRp motifs (Poch et al., 1989, Koonin, 1991, Bruenn, 2003). The 795-position alignment generated using MAFFT appears notably robust in terms of including gaps at only 7 positions other than in the terminal regions, in having 136 positions (17%) that are wholly conserved among the 21 ORF2p sequences included in this comparison, and in having 451 positions in the consensus (57%) that are at least similar among all 21 of the sequences (Fig. S1). RdRp motifs A, B, and C (or IV, V, and VI) are especially easy to spot in the consensus and occur in the usual order: A, 341-shhELDWtKFDRnRP-352; B, 406-hpGMVPSGSLWTGhhsTuhNhhY-426; and C, 445-CAGDDNLT-454 (h, hydrophobic; n, negatively charged; p, polar; s, small; t, turn-like; u, tiny). There are also regions of strong sequence conservation near the C-terminus of ORF2p, seemingly beyond the central region of conserved RdRp motifs (Fig. S1, Fig. 4A), suggesting that another conserved function might be mediated by these C-terminal sequences. A large central portion of the MAFFT alignment is nearly identical with one generated using PROMALS3D, which additionally predicts a consensus secondary structure comprising a mixture of α-helices and β-strands (Fig. S1).

Multiple sequence alignments for ORF1p from proposed and original plant amalgaviruses were also examined in detail for conserved residues. As expected from the pairwise scores (Fig. 2), the 413-position alignment generated using MAFFT shows a much lower degree of conservation than the alignment for ORF2p, including only 1 position (a Gly residue) that is wholly conserved among the 22 ORF1p sequences included in this comparison. The ORF1p alignment nevertheless appears robust in including gaps at only 4 alignment positions besides in the terminal regions and in having 89 alignment positions (22%) at which at least similar residues are found in all 22 of the sequences (Fig. S2). A large central portion of this alignment is nearly identical with one generated using PROMALS3D, which additionally predicts a consensus secondary structure comprising many α-helices and notably no β-strands (Fig. S2). Prediction of predominantly α-helical content for amalgavirus ORF1p has been previously reported (Sabanadzovic et al., 2009, Sabanadzovic et al., 2010, Krupovic et al., 2015). In addition, we newly observed that a central span of 19–46 residues is predicted in all of the different proposed and approved plant amalgaviruses to form an α-helical coiled coil structure (Fig. S2, Fig. 4B), which would be an unusual finding for a viral CP that assembles into an icosahedral particle. This new observation may thus support the suggestion that amalgavirus ORF1p forms some other type of structure, such as a filamentous nucleocapsid (Krupovic et al., 2015) or a more amorphous replication factory matrix (Isogai et al., 2011). Interestingly, too, the ORF1 products from ZbV-Z and unirnaviruses, as well as the RNA2 products from most CTTV-like viruses (all but RHsDRV1; see Table S2 for abbreviations and GenBank numbers), are also predicted to form α-helical coiled coil structures (Fig. S4), suggesting that the non-RdRp proteins from all these clades may share structural and functional characteristics, and possibly a common ancestor. See Discussion for additional considerations in this regard.

The two TSA accessions from *A. cepa* (bulb onion), which we now propose to represent novel plant amalgaviruses (Table 1), were derived respectively from two cultivars, OH1 and DH5225, seeds of which were gifted to us by Dr. Michael J. Havey (USDA-ARS and University of Wisconsin-Madison). Using internal primers designed from these two accessions, we were able to generate RT–PCR amplicons of expected sizes (825–875 bp) from RNA isolated from shoots (OH1) or seeds (DH5225) of these two cultivars. Moreover, upon Sanger sequencing of the amplicons, we found their sequences to be ≥99.5% identical to those of the respective TSA accessions (matching nt 1710–2531 of OH1 and nt 1522–2313 of DH5225). These findings provide further evidence that each of these two *A. cepa* cultivars is persistently infected with the respective amalgavirus.

## 3 Discussion

One question that arises is whether the TSA-derived sequences characterized here (see Table 1) represent transcripts of chromosomal or extrachromosomal, host or viral, origin. In recent years, remnants of many nonretroviral RNA virus genomes have been found integrated in host chromosomes (Chiba et al., 2011, Katzourakis and Gifford, 2010, Taylor and Bruenn, 2009) and, if transcribed, may be detected in transcript-derived databases. In the vast majority of these cases, however, the integrated viral elements are notably fragmented, and their ORFs are disrupted by stop codons and frame-shift mutations. This is notably unlike the case for the TSA-derived sequences listed in Table 1, which approximate the lengths of complete plant amalgavirus genomes and have the expected long ORFs for expressing ORF1p and ORF1+2p. Thus, we conclude that all of the TSA accessions in Table 1 likely represent *bona fide* plant amalgaviruses, which were infecting the respective plants at the times of sampling for transcriptome analyses.

The TSA accession from *C. annuum*, representing putative amalgavirus CaAV1, is notable for lacking a copy of the UUU_CGN consensus motif for +1 PRF in its ORF1–ORF2 overlap region. As noted above, CaAV1 is quite similar to STV in pairwise comparisons (Fig. 2), and indeed their two RdRp sequences approach an identity threshold (65–70%) often used for assigning RNA virus strains to the same or different species. Interestingly, STV is also like CaAV1 in lacking a copy of the UUU_CGN consensus motif for +1 PRF in its ORF1–ORF2 overlap region (Depierreux et al., 2016, Firth et al., 2012), and their respective plants of origin, tomato and pepper, are members of the same taxonomic family and subfamily, *Solanaceae/Solanoideae*, indeed of two closely related tribes, *Solanaceae* and *Capsiceae*, within that subfamily (Särkinen et al., 2013). In an effort to identify an atypical +1 PRF motif in CaAV1, we examined the multiple sequence alignments of both the plus-strand RNA and the full-length ORF2 translation products of the proposed and original plant amalgaviruses (Fig. S3). Based on these alignments, the motif for +1 PRF in CaAV1 is predicted to be CUU_AGU_C (Fig. 1C), where translation of the CUU codon is followed by translation of the GUC codon consequent to +1 PRF. Notably with this motif, the anticodon 3′-GAI (I=inosine) decoding codon CUU (Grosjean et al., 2010) could remain engaged in the ribosomal P site upon forward slippage to codon UUA, including a G:U pair in the first position. Although the +1 shift in STV was previously suggested to occur on motif AGG_CGU_C (see Fig. 1B), based on the RNA alignment (Fig. S3) and other considerations, we now suggest that the +1 PRF motif of STV would be better revised backward by one codon to CUU_AGG_C, making it very similar to CUU_AGU_C in CaAV1 and still allowing P-site anticodon:codon pairing after ribosomal slippage from CUU to UUA (Fig. 1C).

Interestingly, the same heptanucleotide, CUU_AGG_C, is utilized for highly efficient +1 PRF in *Saccharomyces cerevisiae* Ty1, Ty2, and Ty4 elements (Belcourt and Farabaugh, 1990). There, high efficiencies (up to ~40%) depend in part on the low availability in *S. cerevisiae* of the tRNA^Arg^ with anticodon 3′-UCC. In plants, however, this tRNA appears not to be limiting so that frameshifting efficiencies may be much lower, perhaps consistent with the ~1–2% frameshifting efficiencies measured in rabbit reticulocyte lysates for the UUU_CGN influenza A virus shift site seemingly shared by other amalgaviruses (Jagger et al., 2012). Notably, the codon proposed to be in the A site at the onset of frameshifting differs between CaAV1 (AGU, encoding Ser) and STV (AGG, encoding Arg). Similarly, for the sequences with proposed UUU_CGN shift sites, all four CGN arginine codons (corresponding to three tRNA^Arg^ iso-acceptors) are represented. This suggests there may be specific features of CGN and AGN A-site codons, other than simply the availability of the cognate tRNA (and aside from the obvious restrictions at the first codon position, C or A, to permit +1 re-pairing of the P-site tRNA), that favor P-site +1 slippage.

UvNV1 and NoURV1 (Zhang et al., 2014, Zhou et al., 2016) (see Table S2 for abbreviations and GenBank numbers) are two recently described mycoviruses with monosegmented dsRNA genomes that have ORF2 (encoding RdRp) positioned in the +1 frame relative to ORF1. They are related to each other but, according to phylogenetic analyses with RdRp sequences, they are more distantly related to plant amalgaviruses than is amalga-like mycovirus ZbV-Z (e.g., see Fig. 3). Notably, however, both UvNV1 (Zhang et al., 2014) and NoURV1 (this report) have motif UUU_CGA properly positioned in the region of ORF1–ORF2 overlap to be their potential +1 PRF site. Also, the ORF1 translation product of each, which is quite small (172 or 174 aa), is predicted to be predominantly α-helical in secondary structure and to have propensity for coiled coil formation (Fig. S4). Primary sequence conservation across the ORF1 products of plant amalgaviruses, ZbV-Z, and UvNV1 and NoURV1 appears limited. However, with MAFFT (Fig. S2) as well as several other alignment programs, we noted a 100- to 150-aa central region of ORF1p from all these viruses that aligned in three large blocks with no gaps, including across the largely conserved Gly residue and the region with consistently predicted coiled coil propensity (Fig. S2). These findings suggest to us that ORF1p from plant amalgaviruses, ZbV-Z, and UvNV1 and NoURV1 are indeed all homologs, thus presumably sharing a common ancestor.

In our original tblastn search against the TSA database for plants, we found a number of additional accessions with E-value scores between 0.0 and 1e−30, indicative of still strong similarities with the BLV ORF1+2p query. Fourteen of these accessions were from 9 different plant species not represented in Table 1 (*Agropyron cristatum*, *Atractylodes lancea*, *Camellia sinensis*, *Fritillaria cirrhosa*, *Gentiana macrophylla*, *Phalaenopsis aphrodite*, *Prosopis alba*, *Reaumuria trigyna*, and *Solanum melongena*); however, none of them were >1898 nt in length (Table S2), such that they do not approach the genome lengths of plant amalgaviruses. When used in a subsequent blastx search against the full NR database, each of these 14 TSA accessions scored most highly nonetheless with one of the four originally characterized plant amalgaviruses (E-value scores ≤8e−32). Moreover, upon examining their sequences, we found that one reading frame of each accession approximates an end-to-end ORF, the translated product of which in a PSI-BLAST search showed protein sequence similarity across approximately its full length with at least one of the original amalgaviruses (E-value scores ≤4e−38). We therefore consider it likely that the TSA accessions listed in Table S3 represent partially determined sequences of yet other *bona fide* amalgaviruses, which were infecting these additional plant species at the times of sampling for transcriptome analyses. TSA accessions with E-value scores >1e−30 in the initial tblastn search may also hold interesting findings but were outside the focus of this study.

The TSA accessions and SRA data sets used in this study are associated with peer-reviewed publications in some cases (Czaban et al., 2015, Duangjit et al., 2013, Farrell et al., 2014, Gould et al., 2015, Khalil et al., 2015), but not in others. Moreover, none of the TSA accessions are currently annotated to indicate their viral origins. This lack of annotation will make it difficult for many investigators to locate these sequences for inclusion in phylogenetic analyses or other comparisons. We have therefore been attempting, though without success to date, to deposit the newly proposed plant amalgavirus sequences summarized in Table 1 as Third-Party Annotations at GenBank, in an effort to make them easier to locate via their metadata. A more routine procedure for encouraging and accepting such new deposits based on sequence data previously made public at NCBI – especially those sequence data in the TSA, SRA, and other databases that have been rapidly expanding consequent to next-generation sequencing methods – seems likely to be of broad benefit.

## 4 Materials and methods

All database searches were performed with the indicated programs as implemented with defaults at http://blast.ncbi.nlm.nih.gov/Blast.cgi. Searches of the TSA database with protein sequence queries deduced from nucleotide sequences were performed using tblastn. Searches of the SRA database with nucleotide sequence queries were performed using discontiguous megablast. For the TSA and SRA searches, default settings were sometimes altered to allow larger numbers of target sequences (>100) to be displayed. Searches of the NR database with nucleotide sequence queries or with protein sequence queries deduced from nucleotide sequences were performed using blastx or PSI-BLAST, respectively.

Given the incomplete protein-coding regions in some of the amalgavirus-like TSA accessions that we first discovered (GAMH01005363, GAYX01076418, GBIE01028534, GBXZ01009138, GCJW01039808, GEAC01063629, and GECO01025317; Table 1, top), we accessed the SRA data sets from each of those transcriptome projects and in discontiguous megablast searches found reads that mapped to each of the original TSA accessions. We then used CAP3 (Huang and Madan, 1999) or CLC Genomics Workbench 8 (Qiagen) to assemble contigs that were compared with the TSA sequence. In the cases of TSA accessions GAYX01076418, GBXZ01009138, GCJW01039808, GEAC01063629, and GECO01025317, we were able to extend the original sequence at one or both termini in this manner. We reiteratively repeated this process to add new SRA accessions to each extending terminus until newly matching accessions were no longer found. The SRA data sets searched for each of the originally truncated TSA sequences were: GAMH01005363, SRX329048 and SRX329051; GAYX01076418, SRX670823–SRX670828; GBIE01028534, SRX1733822–SRX1733825; GBXZ01009138, SRX757539; GCJW01039808, DRX000652–DRX000659; GEAC01063629, SRX1374921–SRX1374944; and GECO01025317, SRX1427152–SRX1427157.

ORFs were identified in nucleotide sequences using EMBOSS getorf as implemented at http://www.bioinformatics.nl/emboss-explorer/ or ExPASy Translate as implemented at http://web.expasy.org/translate/. Multiple sequence alignments of RNA or protein sequences were performed using MAFFT 7.2 (L-INS-i) (Katoh and Standley, 2013) as implemented with defaults at http://mafft.cbrc.jp/alignment/server/. Multiple sequence alignments accompanied by secondary structure predictions were obtained using PROMALS3D (Pei and Grishin, 2014) as implemented with defaults at http://prodata.swmed.edu/promals3d/promals3d.php. Global pairwise alignments of protein sequences were performed using Needle (Needleman and Wunsch, 1970) or Needleall as implemented with defaults at http://www.bioinformatics.nl/emboss-explorer/. Average degree of conservation along a multiple sequence alignment was plotted using EMBOSS: plotcon as implemented with defaults (except window size=10) at http://www.bioinformatics.nl/emboss-explorer/. Coiled coil predictions were obtained using MARCOIL or COILS/PCOILS (Lupas, 1996) as implemented with defaults at http://toolkit.tuebingen.mpg.de/.

Phylogenetic relationships were determined using PhyML 3.0 (Guindon et al., 2010) as implemented at http://www.hiv.lanl.gov/content/sequence/PHYML/interface.html with the following parameters differing from the defaults: Sequence type/model, Amino acids/LG or rtREV; Proportion of invariable sites, estimated from data; Gamma shape parameter, estimated from data; Starting tree(s) optimization, Tree topology and Branch length; Tree improvement, Best of NNI and SPR; Branch support, Approximate Likelihood Ratio Test (aLRT), SH-like supports. The results in Newick format were then submitted to TreeDyn 198.3 as implemented at http://www.phylogeny.fr/ for displaying branch support values in % and collapsing branches with lower support values. The output in Newick format was then opened in FigTree v1.4.0 (downloaded from http://tree.bio.ed.ac.uk/software/figtree/) for refining the phylogram for presentation.

Table S2 lists abbreviations and GenBank accession numbers for nucleotide sequences of other dsRNA viruses included in this study besides those in Table 1 and Table S1. The ORF2p (RdRp) sequences used for multiple sequence alignments or global pairwise alignments began with the first residue after the site of predicted PRF in ORF2 for plant amalgaviruses, ZbV-Z, unirnaviruses, and UvNV1 and NoURV1, and with the first in-frame Met in the RdRp-encoding ORF for CTTV-like viruses and partitiviruses; all ORF2p (RdRp) sequences ended with the last residue before the ORF2 stop codon unless otherwise noted in the Fig. 2 legend. The ORF1p sequences used for global pairwise alignments began with the first in-frame Met in ORF1 for all viruses and ended with the last residue before the ORF1 stop codon unless otherwise noted in the Fig. 2 legend.

## Figures and Tables

**Table 1:** Newly proposed (top) and original (bottom) plant amalgaviruses.

Putative host species (cultivar)	GenBank accession no.	Amalgavirus (abbrev.)	Length (bp)^a^	ORF1p (aa)^b^	ORF2p (aa)^c^	ORF1+2p (aa)^d^
*Allium cepa* (OH1)	GAAO01011981^e^	AcAV1	3453	391	779	1057
*Allium cepa* (DH5225)	GAAN01008476^e^	AcAV2	3453	390	787	1065
*Anthoxanthum odoratum*	GBIE01024896^e^	AoAV1	3356	382	783	1056
*Anthoxanthum odoratum*	GBIE01028534^e^	AoAV2	(2971)	(388)	(716)	(989)
*Camellia oleifera* (Xianglin4)	GEFY01004381	CoAV1	3333	398	774	1066
*Capsicum annuum* (CM334)	JW101175	CaAV1	3478	375	774	1062
*Cleome droserifolia*	GDRJ01026949	CdAV1	3443	402	774	1070
*Erigeron breviscapus*	GDQF01098448	EbAV1	3433	384	784	1049
*Erigeron breviscapus*	GDQF01120453	EbAV2	3408	386	785	1054
*Festuca pratensis* (Laura)	GBXZ01049574^e^	FpAV1	3412	382	784	1057
*Festuca pratensis* (Laura)	GBXZ01002308^e^	FpAV2	3411	385	774	1053
*Festuca pratensis* (Laura)	GBXZ01009138^e^	FpAV3	(3288)	385	(768)	(1047)
			3381^f^	385	769	1048
*Gevuina avellana* (Mol.)	GEAC01063629	GaAV1	(2793)	(228)	774	(896)
			3401^f^	403	774	1071
*Lolium perenne* (P226/135/16)	GAYX01076418^e^	LpAV1	(3296)	385	(770)	(1049)
			3373^f^	385	769	1048
*Medicago sativa*	GAFF01077243	MsAV1	3423	394	772	1058
*Phalaenopsis equestris*	GDHJ01028335	PeAV1	3394	384	781	1059
*Pinus patula*	GECO01025317	PpAV1	(3015)	(322)	777	(1003)
			(3186)^f^	(365)	777	(1046)
*Salicornia europaea*	GAMH01005363	SeAV1	(2798)	382	(613)	(880)
*Secale cereale*	GCJW01039808^e^	ScAV1	(2851)	382	(633)	(916)
			3412^f^	398	781	1064

*Blueberry latent virus*	HM029246^e^	BLV	3431	375	789	1054
*Rhododendron virus A*	HQ128706^e^	RHV-A	3427	404	777	1077
*Southern tomato virus*	EF442780^e^	STV	3437	377	774	1062
*Vicia cryptic virus M*	EU371896^e^	VCV-M	3434	394	771	1057

a Nucleotide sequences that appear to be truncated at one or both ends have their lengths listed in parentheses.

b For apparently full-length ORF1 translation products, the lengths are calculated from the first in-frame Met residue to the first in-frame stop codon. For ORF1 translation products that appear to be truncated at one or both ends, the lengths are calculated to the termini and are listed in parentheses.

c For apparently full-length ORF2 translation products, the lengths are calculated from the first residue following the proposed +1 PRF site to the first in-frame stop codon. For ORF2 translation products that appear to be truncated at the C-terminal end, the lengths are calculated from the first residue following the proposed +1 PRF site to the C-terminus and are listed in parentheses.

d For apparently full-length ORF1+2 translation products, the lengths are calculated from the first in-frame Met residue in ORF1p to the first in-frame stop codon in ORF2p, taking into account the proposed +1 PRF site. For ORF1+2 translation products that appear to be truncated at one or both ends, the lengths are calculated to the respective termini, taking into account the proposed +1 PRF site.

e Sequences for which peer-reviewed papers are also available, as indicated in the text.

f Sequences that were extended by reassembling contigs from SRA entries (see text and Table S1).

## Footnotes

Appendix A Supplementary data associated with this article can be found in the online version at http://dx.doi.org/10.1016/j.virol.2016.07.002.
