# Supplementary figures and images for: A +1 ribosomal frameshifting motif prevalent among plant amalgaviruses
Source: Virology. 2016 Nov;498:201–8. doi: 10.1016/j.virol.2016.07.002 (PMC5052127; doi:10.1016/j.virol.2016.07.002)

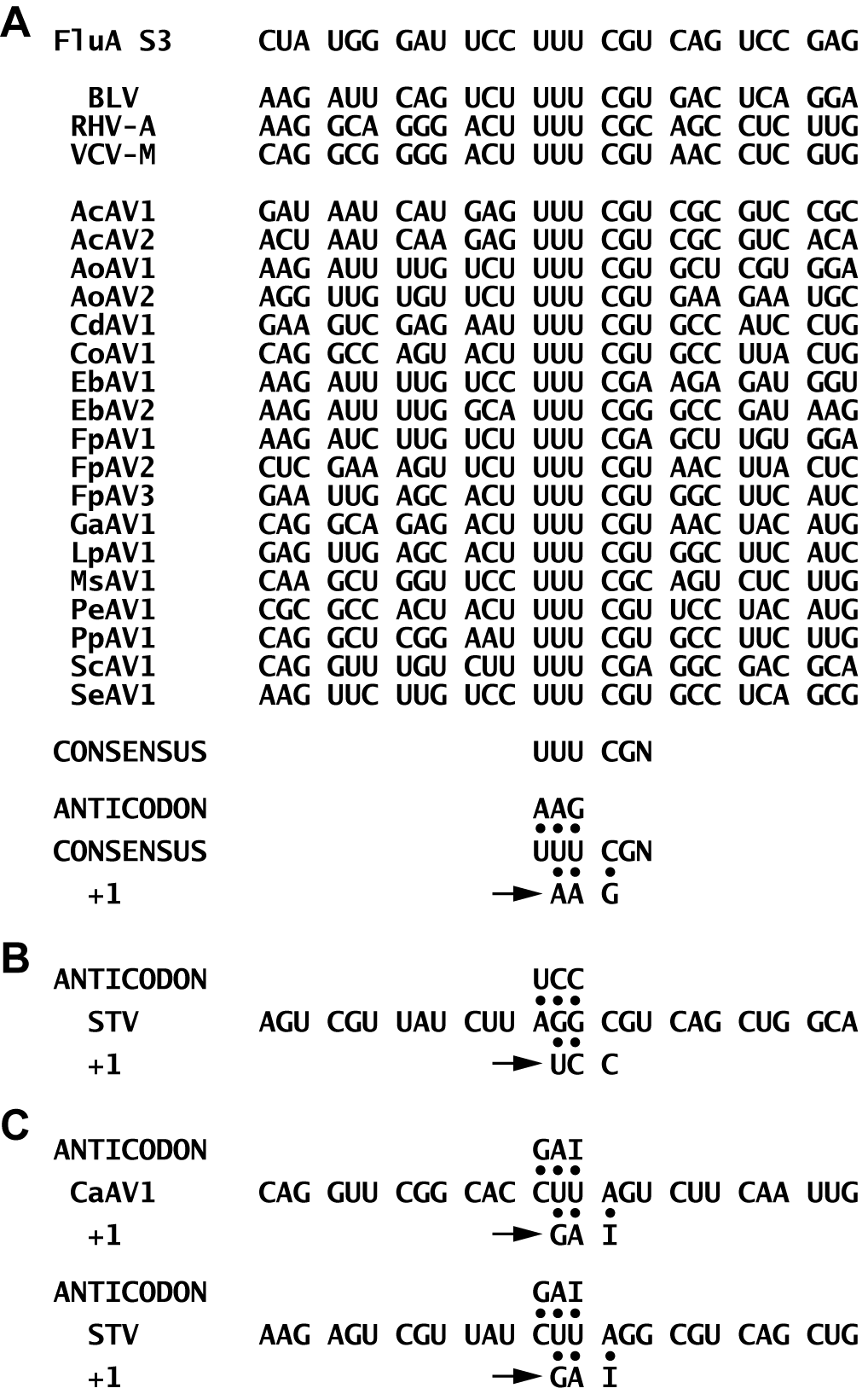

Supplement: Supplementary file 2 — Supplementary material [file mmc2.zip › NewAmalga-RevFig1.tif]

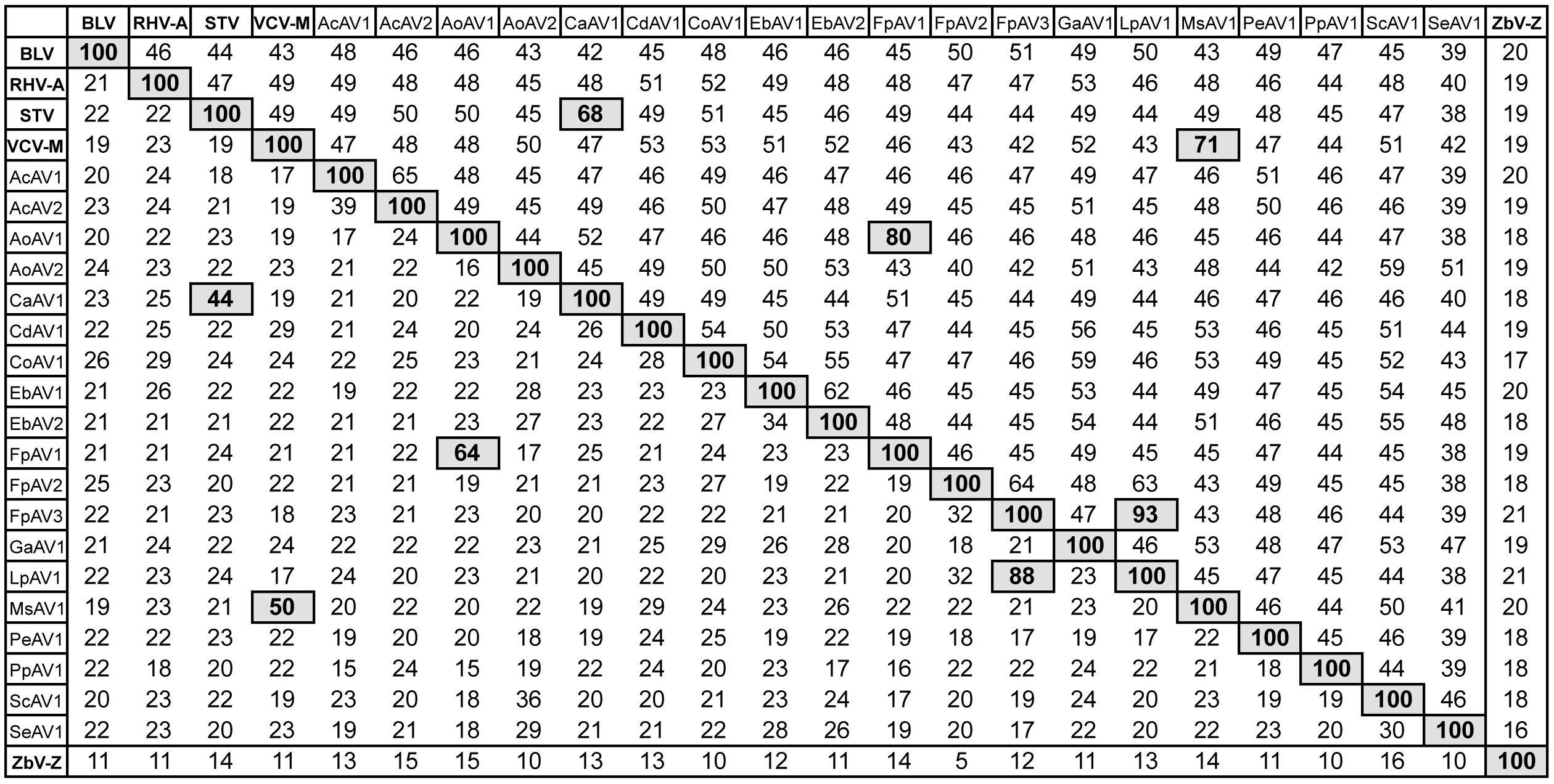

Supplement: Supplementary file 3 — Supplementary material [file mmc3.zip › newAmalga-RevFig2.tif]

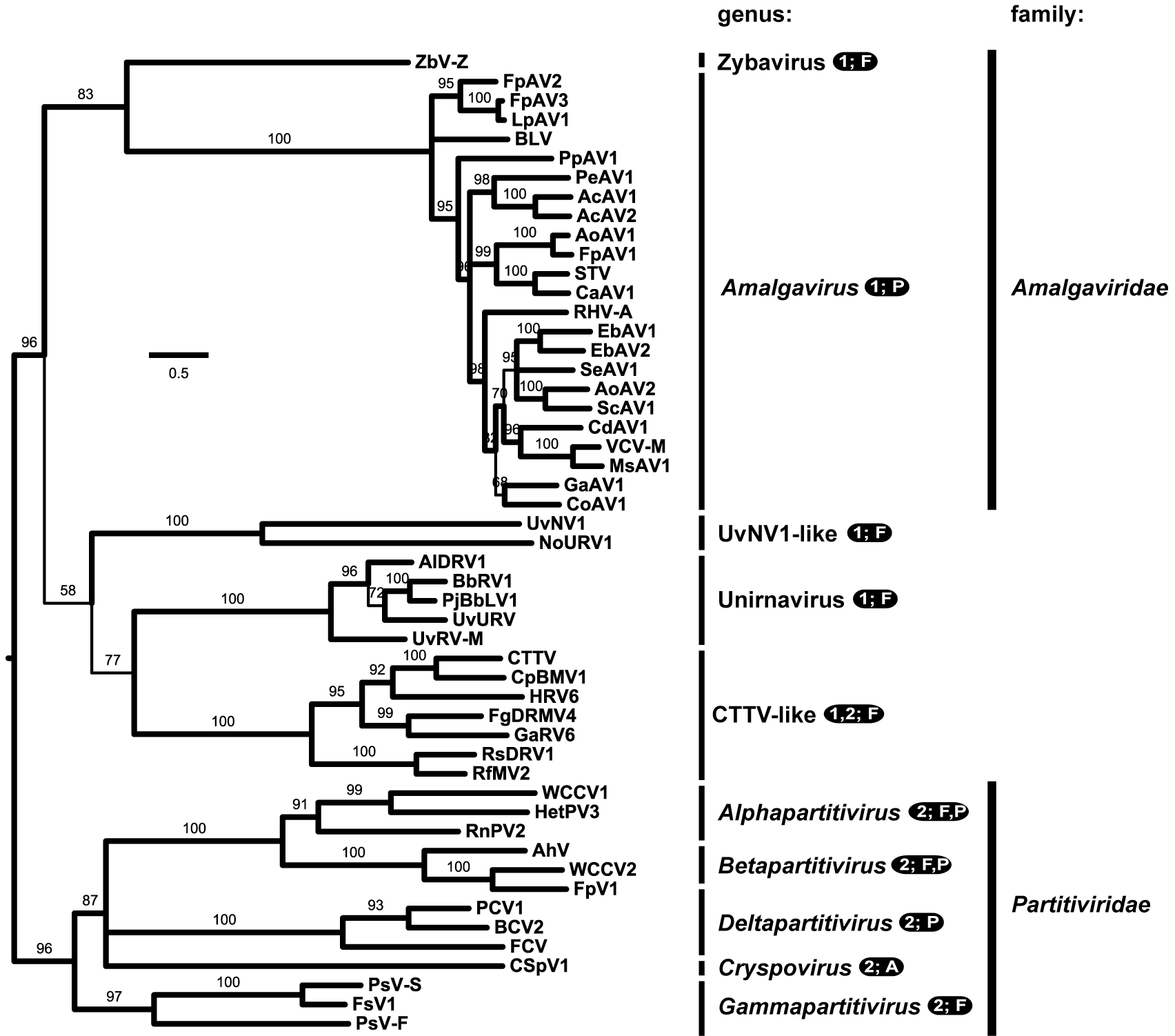

Supplement: Supplementary file 4 — Supplementary material [file mmc4.zip › NewAmalga-RevFig3.tif]

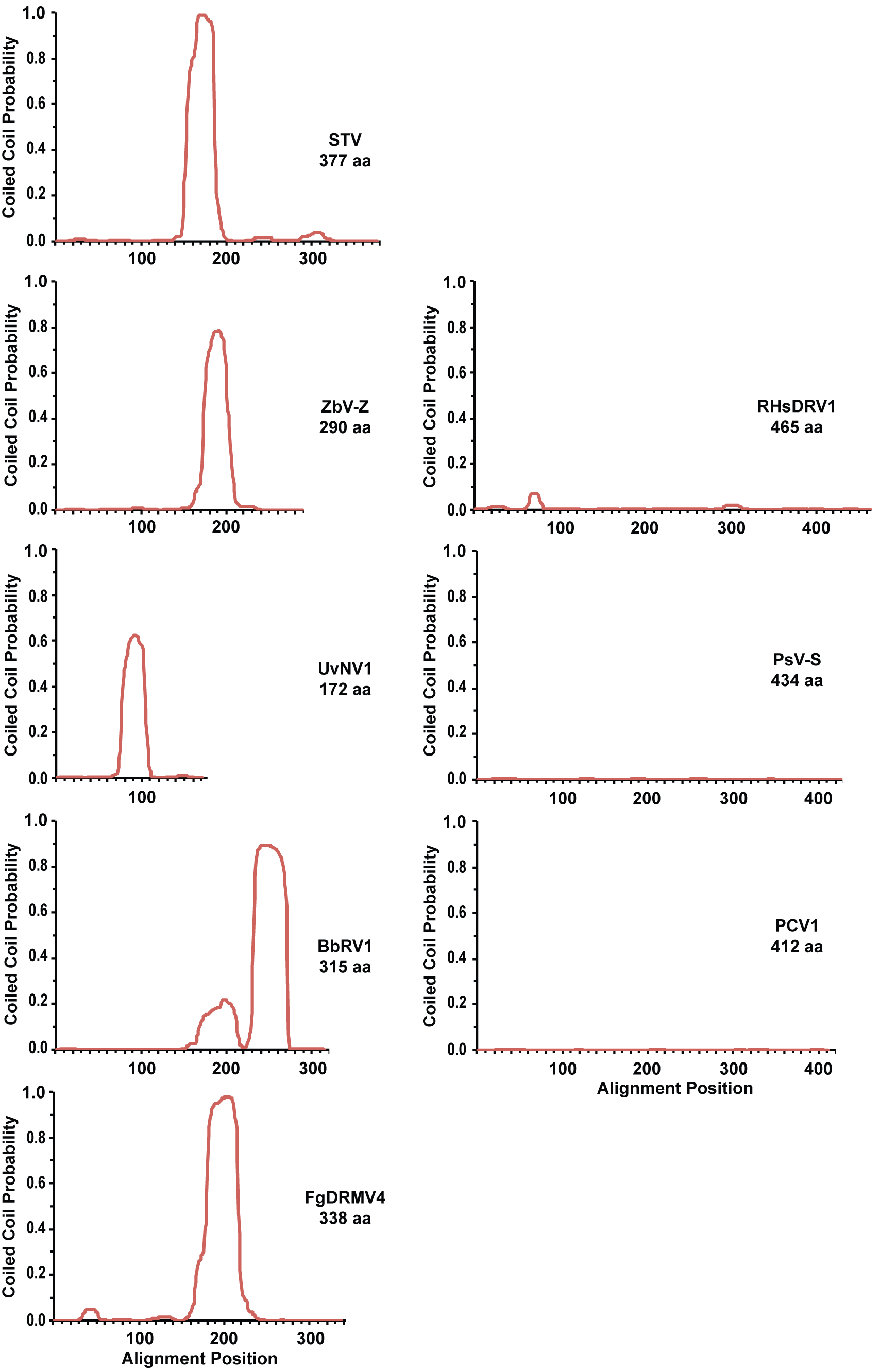

Supplement: Supplementary file 5 — Supplementary material [file mmc5.zip › NewAmalga-RevFigS4.tif]
