# Supplementary material for: A +1 ribosomal frameshifting motif prevalent among plant amalgaviruses
Source: Virology. 2016 Nov;498:201–8. doi: 10.1016/j.virol.2016.07.002 (PMC5052127; doi:10.1016/j.virol.2016.07.002)
Supplement: Supplementary file 6 — Supplementary material [file mmc6.doc]

**Table S1**

Sequence reassembly information for originally truncated TSA accessions

-------------------------------------------------------------------------------------------------------------------------------------------------------

Proposed GenBank Sequencing Total SRA Matching SRA Mapped Positional coverage:

amalgavirus accession no. format reads^a^ reads (% of total)^b^ SRA reads^c^ mean ± SD (range)^d^

-------------------------------------------------------------------------------------------------------------------------------------------------------

FpAV3 GBXZ01009138 Illumina 77,855,198 3993 (0.0047%) 3665 96 ± 38 (1–258)

GaAV1 GEAC01063629 Illumina 202,229,984 677 (0.0003%) 677 17 ± 10 (1–48)

LpAV1 GAYX01076418 Illumina 87,078,920 3450 (0.0040%) 3431 101 ± 48 (1–234)

PpAV1 GECO01025317 Illumina 440,837,728 6417 (0.0015%) 6333 195 ± 126 (4–607)

ScAV1 GCJW01039808 454 5,693,480 69 (0.0012%) 57 6 ± 4 (1–18)

-------------------------------------------------------------------------------------------------------------------------------------------------------

^a^ No. of individual reads from the corresponding SRA file(s) that were searched by discontiguous megablast for each transcript.

^b^ No. of individual reads from the corresponding SRA file(s) that scored as matches by discontiguous megablast for each final reassembled transcript.

^c^ No. of individual reads that were mapped to the final reassembled transcript by CLC Genomics Workbench 8.0.

^d^ Results of coverage analysis after read mapping by CLC Genomics Workbench 8.0. The regions of each assembly covered by single reads were restricted to the 5´ and 3´ termini, outside the central protein-coding region of each assembly.
