# Supplementary material for: A +1 ribosomal frameshifting motif prevalent among plant amalgaviruses
Source: Virology. 2016 Nov;498:201–8. doi: 10.1016/j.virol.2016.07.002 (PMC5052127; doi:10.1016/j.virol.2016.07.002)
Supplement: Supplementary file 7 — Supplementary material [file mmc7.doc]

**Table S2**

GenBank accession numbers for the nucleotide sequences of mono- and bisegmented dsRNA viruses included for analysis in this report (in addition to those in Tables 1 and S1)

--------------------------------------------------------------------------------------------------------------------

Virus (alphabetical) Abbrev. GenBank no.

--------------------------------------------------------------------------------------------------------------------

Alternaria longipes dsRNA virus 1 AlDRV1 KJ817371

Atkinsonella hypoxylon virus AhV L39125, L39126^a^

Beauveria bassiana RNA virus 1 BbRV1 LN610699

Beet cryptic virus 2 BCV2 HM560703, HM560702

Cryphonectria parasitica bipartite mycovirus 1 CpBPMV1 KC549809, KC549810

Cryptosporidium parvum virus 1 CSpV1 U95995, U95996

Curvularia thermal tolerance virus CTTV EF120984, EF120985

Fig cryptic virus FCV FR687854, FR687855

Fusarium graminearum dsRNA mycovirus 4 FgDRMV4 GQ140627, GQ140628

Fusarium poae virus 1 FpV1 AF047013, AF015924

Fusarium solani virus 1 FsV1 D55668, D55669

Gremmeniella abietina RNA virus 6 GaRV6 KJ742567

Heterobasidion partitivirus 3 HetPV3 FJ816271, FJ816272

Heterobasidion RNA virus 6 HRV6 KF551895

Nigrospora oryzae unassigned RNA virus 1 NoURV1 KT258976

Penicillium janczewskii B. bassiana-like virus 1 PjBbLV1 KT601106

Penicillium stoloniferum virus F PsV-F AY738336, AY738337

Penicillium stoloniferum virus S PsV-S AY156521, AY156522

Pepper cryptic virus 1 PCV1 JN117276, JN117277

Rhizoctonia fumigata mycovirus RfMV2 KP209316, KP209317

Rhizoctonia solani dsRNA virus 1 RHsDRV1 JX976612, JX976613

Rosellinia necatrix partitivirus 2 RnPV2 AB569997, KJ605398

Ustilaginoidea virens RNA virus M UvRV-M KJ101567

Ustilaginoidea virens unassigned RNA virus UvURV KR106133

Ustilaginoidea virens nonsegmented virus 1 UvNV1 KJ605397

White clover cryptic virus 1 WCCV1 AY705784, AY705785

White clover cryptic virus 2 WCCV2 JX971976, JX971977

Zygosaccharomyces bailii virus Z ZbV-Z KU200450

--------------------------------------------------------------------------------------------------------------------

^a^ For viruses with two numbers listed, the first is for the RdRp-encoding genome segment
