# Supplementary material for: A +1 ribosomal frameshifting motif prevalent among plant amalgaviruses
Source: Virology. 2016 Nov;498:201–8. doi: 10.1016/j.virol.2016.07.002 (PMC5052127; doi:10.1016/j.virol.2016.07.002)
Supplement: Supplementary file 8 — Supplementary material [file mmc8.doc]

**Table S3**

Additional top-scoring hits from the initial tblastn search of the TSA database for plants, using BLV ORF1+2p as query

---------------------------------------------------------------------------------------------------------

Putative host species name ^a^ GenBank Length Blastx top hit

accession no. (bp) (amalgavirus, E-value) ^b^

---------------------------------------------------------------------------------------------------------

*Agropyron cristatum* GBAU01007640 1325 RHV-A, 2e−140

*Atractylodes lancea* GEFZ01018041 686 BLV, 1e−86

*Camellia sinensis v. sinensis*  GBKQ01025649 1898 RHV-A, 0.0

*Camellia sinensis v. sinensis* GAAC01006570 444 STV, 2e−48

*Camellia sinensis v. sinensis*  GAAC01041325 415 RHV-A, 9e−38

*Fritillaria cirrhosa*  GAGV01022846 460 STV, 2e−57

*Gentiana macrophylla* GAJR01024778 345 STV, 1e−42

*Phalaenopsis aphrodite* JI639011 486 BLV, 2e−42

*Phalaenopsis aphrodite* JI659538 365 STV, 1e−43

*Phalaenopsis aphrodite* JI653329 250 BLV, 8e−32

*Prosopis alba* GAOO01021648 513 STV, 2e−72

*Reaumuria trigyna* JR242770 865 RHV-A, 8e−108

*Reaumuria trigyna* JR258007 550 BLV, 1e−61

*Solanum melongena* GBGZ01101753 451 STV, 4e−57

---------------------------------------------------------------------------------------------------------

^a^ See text for additional explanations of this table; only hits from the TSA database with initial E-values <1e-30, and from plant species not already represented in Table 1, are shown.

^b^ The amalgavirus representing the top hit in a subsequent blastx search of the full NR database is indicated (abbrev.), along with its E-value score.
