# Supplementary material for: A +1 ribosomal frameshifting motif prevalent among plant amalgaviruses
Source: Virology. 2016 Nov;498:201–8. doi: 10.1016/j.virol.2016.07.002 (PMC5052127; doi:10.1016/j.virol.2016.07.002)
Supplement: Supplementary file 9 — Supplementary material [file mmc9.doc]

**Supplementary** **Figure Legends**

**Fig. S1.** MAFFT alignment, ORF2p (RdRp). ORF2p (post-frameshift) sequences from the indicated amalgaviruses were aligned using MAFFT. The alignment was then reformatted using MView as implemented at http://www.ebi.ac.uk/Tools/msa/mview/. Consensus (cons) amino acids have been assigned to classes according to MView convention: a, aromatic; c, charged; h, hydrophobic; l, aliphatic; o, alcohol; p, polar; s, small; t, turnlike; u, tiny; +, positively charged; and -, negatively charged. Gray shading: gaps. Red lettering: consensus positions with no more than 4 different amino acids in the different sequences. Light cyan shading: RdRp motifs A, B, and C. PROMALS3D: secondary structure predictions at each position (α-helix or β-strand) across a large central region in which the MAFFT and PROMALS3D alignments are nearly identical. The C-terminally truncated ORF2p sequences for AoV2 and SeAV1 (see Table 1) were omitted from this analysis.

**Fig. S2.** MAFFT alignment, ORF1p. ORF1p sequences from the indicated amalgaviruses were handled, and the results labeled, in the same ways as for the ORF2p sequences in Fig. S1. Yellow-green shading: regions of coiled coil prediction (>50% probability) by MARCOIL or COILS (averaging windows, 14, 21, or 28 residues); the apparent register of the heptad repeat (*abcdefg*; *a* and *d*, hydrophobic) in a portion of the central, conserved region with predicted coiled coil propensity is labeled at bottom. The N-terminally truncated ORF1p sequence for PpAV1 (see Table 1) was omitted from these analyses. A separate MAFFT alignment, to which sequences from ZbV-Z, UvNV1, and NoURV1 were added to those of the plant amalgaviruses, identified three blocks of aligned sequences without gaps as shown here, the middle of which corresponded with the central, conserved region of predicted coiled coil propensity in amalgaviruses as well as in the 3 added viruses (darker green shading).

**Fig. S3.** MAFFT alignment, RNA: +1 PRF motifs. (A) Plus-strand RNA sequences from the indicated amalgaviruses were aligned using MAFFT. A portion of the alignment encompassing the proposed +1 PRF motif in each sequence (orange or green text) is shown. Notably, the alignment includes no gaps in this region, and all of the proposed +1 PRF motifs align at only 3 different positions within a span of only 50 nt. The proposed motifs for CaAV1 and STV are in green text because they represent variants to the consensus; the motif previously proposed for STV (shifted forward by 1 codon) is underlined along with the corresponding sequence from CaAV1. Cyan lettering: stop codons flanking the upstream end of ORF2 (not present for all sequences in the nucleotide region shown here). There are no stop codons flanking the downstream end of ORF1 in the region shown). Number at end of each line: nucleotide position of the last base shown; for sequences that are 5´-truncated with regard to the protein coding region, this number is shown in parentheses. (B) Amino acid translation is shown for ORF2 of each nucleotide sequence. Gray or black text: amino acids respectively before or after the site of the proposed +1 PRF. Val, translated from GUN codons, occurs in 16 of the 23 sequences as the first amino acid encoded after the proposed +1 PRF.

**Fig. S4.** Coiled coil predictions, ORF1p. The indicated ORF1p sequences were analyzed using MARCOIL. STV represents plant amalgaviruses, UvNV1 represents the emerging taxon that also contains NoURV1, BbRV1 represents unirnaviruses, FgDRMV4 represents most CTTV-like viruses, RHsDRV1 represents a CTTV-like virus that lacks predicted coiled coil propensity, and PCV1 and PsV-S represent two genera of partitiviruses. The X-axis of each panel is to the same scale.
